# Use of pregnancy personalised follow-up in case of maternal social vulnerability to reduce prematurity and neonatal morbidity

**DOI:** 10.1186/s12884-023-05604-7

**Published:** 2023-04-26

**Authors:** Simon Crequit, Gregory Bierry, Perbellini Maria, Sakina Bouali, Adelaïde Dupre La Tour, Naima Sgihouar, Bruno Renevier

**Affiliations:** 1 Service de Gynécologie Obstétrique, Centre Hospitalier Intercommunal de Montreuil, 56 Boulevard de la Boissière, 93100 Montfermeil, France; 2 Responsable de L’Unité de Recherche Clinique / GHT Grand Paris Nord Est, GHI Raincy Montfermeil, 10 Rue du Général Leclerc, 93370 Montfermeil, France

**Keywords:** Social vulnerability, Social deprivation, Neonatal outcomes, Neonatal morbidity, SGA, Small for gestational age, Pregnancy outcomes

## Abstract

**Background:**

Social deprivation is a major risk factor of adverse pregnancy outcomes. Yet, there is few studies evaluating interventions aiming at reducing the impact of social vulnerability on pregnancy outcomes.

**Objective:**

To compare pregnancy outcomes between patients that received personalized pregnancy follow-up (PPFU) to address social vulnerability versus standard care.

**Methods:**

Retrospective comparative cohort in a single institution between 2020 and 2021. A total of 3958 women with social vulnerability that delivered a singleton after 14 gestational weeks were included, within which 686 patients had a PPFU. Social vulnerability was defined by the presence of at least one of the following characteristics: social isolation, poor or insecure housing conditions, no work-related household income, and absence of standard health insurance (these four variables were combined as a social deprivation index (SDI)), recent immigration (< 12 month), interpersonal violence during pregnancy, being handicaped or minor, addiction during pregnancy. Maternal characteristics and pregnancy outcomes were compared between patients that received PPFU versus standard care. The associations between poor pregnancy outcomes (premature birth before 37 gestational weeks (GW), premature birth before 34 GW, small for gestational age (SGA) and PPFU were tested using multivariate logistic regression and propensity score matching.

**Results:**

After adjustment on SDI, maternal age, parity, body mass index, maternal origin and both high medical and obstetrical risk level before pregnancy, PPFU was an independent protective factor of premature birth before 37 gestational weeks (GW) (aOR = 0.63, 95%CI[0.46–0.86]). The result was similar for premature birth before 34 GW (aOR = 0.53, 95%CI [0.34–0.79]). There was no association between PPFU and SGA (aOR = 1.06, 95%CI [0.86 – 1.30]). Propensity score adjusted (PSa) OR for PPFU using the same variables unveiled similar results, PSaOR = 0.63, 95%CI[0.46–0.86] for premature birth before 37 GW, PSaOR = 0.52, 95%CI [0.34–0.78] for premature birth before 34 GW and PSaOR = 1.07, 95%CI [0.86 – 1.33] for SGA.

**Conclusions:**

This work suggests that PPFU improves pregnancy outcomes and emphasizes that the detection of social vulnerability during pregnancy is a major health issue.

**Supplementary Information:**

The online version contains supplementary material available at 10.1186/s12884-023-05604-7.

## Background

Since the 80 s, social deprivation has been demonstrated to be a major contributor of inequalities in health [1]. It is now well established that socially deprived women experience more pregnancy complications with an increased risk small for gestational age newborn [2], stillbirth [2, 3], premature birth [4–9], extremely premature birth (both spontaneous and induced [10, 11]). Therefore, neonatal morbidity and mortality are more frequent in socially deprived women [12, 13]. Several works aiming at explaining these discrepancies in term of adverse pregnancy outcomes reported that socially deprived women do not use properly prenatal care [14, 15]. Indeed, vulnerable women seems to address their pregnancy follow-up late, with a lower number of pregnancy consultations and often don’t realize the appropriate number of screening ultrasound [16–18]. Therefore, it seems relevant to detect social deprivation in order to improve perinatal care use and reduce the impact of social vulnerability on pregnancy outcomes. Yet, studies aiming at evaluating the impact of measures to improve the perinatal care use for deprived women are rare. Three studies from the US have demonstrated that the detection of social vulnerability and an appropriate follow-up regarding this latter allowed to reduce stress factors such as interpersonal violence, addiction during pregnancy and improved perinatal care use in socially deprived women [19]. Moreover, two of these studies demonstrated that the companionship of these patients reduced the rate of SGA new-born [20] and premature birth in some sub-groups [21]. Consequently, the finding of new interventions to address social vulnerability is a major health issue. The aim of this study is to evaluate whether a personalized pregnancy follow-up (PPFU) to ease the access to both prenatal care and relevant interventions regarding the patient’s vulnerability would improve neonatal morbidity in case of maternal social vulnerability.

## Material and methods

### Study population

This study is a retrospective comparative cohort between patients that received PPFU versus standard care in case of maternal social vulnerability.

Maternal social vulnerability was defined as the presence of at least one of the following social vulnerabilities: social isolation, poor or insecure housing conditions, no work-related household income, absence of standard health insurance, recent immigration (< 12 month), linguistic barrier, interpersonal violence during pregnancy, being handicapped or minor, addiction during or before pregnancy.

The PPFU unit was implemented in the maternity unit at study in 2017 by the financial help of the local health administration (Agence Régionale de la Santé Île-de-France). It aims at improving maternal pregnancy follow-up in case of social vulnerability to decrease both maternal and neonatal morbidity. It consists of a multidisciplinary unit including midwifes, obstetricians, social workers, psychologists and psychiatrists. The PPFU trains all the professionals of the maternity unit in the detection of social vulnerability so that the detected patients can benefit from a PPFU. The PPFU unit takes care of the consultation booking (both medical and ultrasound appointments) and propose to the included patient a personalized follow-up regarding the relevant needs, (social worker, psychologist, addiction therapist etc.…) that are highlighted by a dedicated multidisciplinary staff. The training of all the professionals of the maternity unit allowed to have access to thorough data regarding social vulnerabilities in the patient’s medical folders.

Standard care in the maternity unit at study is based on the national health authority (Haute Autorité de Santé) recommendations [22], which consists for low-risk pregnancies in 7 consultations performed by midwives and three screening ultrasounds (first trimester, second trimester and third trimester of pregnancy). In case of pregnancy complications or high medical risk level at the beginning of pregnancy, the consultations were performed by obstetricians.

Inclusion criteria in the present study were the combination of the following factors: presence of maternal social vulnerability and a singleton delivery in cephalic presentation after 14 gestational weeks.

Exclusion criteria were one of the following: the absence of maternal social vulnerability, multiple pregnancy, delivery before 14 gestational weeks or non-cephalic presentation at delivery.

Patients’ allocation to PFFU was performed as follow: when maternal social vulnerability was detected during pregnancy follow-up, the practitioner on duty should send a request to the PPFU for inclusion. The PPFU discusses the folders in a multidisciplinary staff and contacts the patients for consent. If the patient accepts, she is included in the PPFU that will provide a personalized pregnancy follow-up.

Patients with maternal social vulnerability that refused PPFU inclusion or that were not reported to the PPFU by the practitioner were included in the standard care group.

Using birth records, a total of 7643 patients who delivered a singleton in cephalic presentation after 14 weeks of gestation were identified between January 2020 and December 2021, in a single tertiary care maternity unit (CHI-Montreuil). Patients that presented maternal social vulnerability (*N* = 3958) were included in the study: 686 patients in the PPFU group that were compared with 3272 patients in the standard care group.

### Collected data

Maternal, pregnancy, labor, delivery, and neonatal characteristics were collected from patient’s computerized pregnancy folder whose content was checked after each delivery in a multidisciplinary staff. Social vulnerabilities were defined as follow: social isolation (absence of partner), Poor or insecure housing condition (no rented nor owned housing), no work-related household income (the woman’s household income came from public assistance, relatives, friends, or a charity), No permanent health care insurance (Couverture Maladie Universelle, CMU) or illegal status (Aide Médicale d’Etat, AME)). These four variables were combined as a quantitative score called the social deprivation index. This latter was developed from the national French survey of 2010 and has been demonstrated to be relevant in classifying the degree of social deprivation in the French population [23].

Recent immigration was defined by an immigration within France < 12 months, linguistic barrier was defined by the need of an interpret during patient follow-up, interpersonal violence during pregnancy was defined as interpersonal violence during pregnancy requiring establishment of protective measures, addictions (before and during pregnancy) were defined by tobacco, alcohol, cannabis, cocaine derived drug or morphine derive drug use, unwanted pregnancy was defined as an unplanned pregnancy that was not desired by the mother.

Inadequate prenatal care use (PCU) was implemented the same way as a former French cohort study of social deprivation [15]: pregnancy follow up began after 12 weeks of gestation, or if it included less than 50% of the number of prenatal visits expected according to duration of pregnancy, or if the first-trimester ultrasound examination or both the second- and third- trimester examinations were missing.

Psychologic follow-up was performed in case of pregnancy related anxiety, depressive symptoms, or patient request for a psychologist follow-up.

Psychiatrist follow-up was performed in case of major depressive disorder, bipolar disorder, post-traumatic stress disorder or Schizophrenia.

Gestational weight gain (GWG) was calculated by subtracting the last measured weight before delivery with the maternal weight before pregnancy. GWG was considered adequate if it was ≥ 1.22 kg per month and ≤ 1.77 kg per month for women presenting a BMI < 30 kg/m^2^ (total pregnancy intake between 11 and 16 kg) [24]. GWG was considered adequate if it was ≥ 0.55 kg per month and ≤ 0.75 kg per month for women presenting a BMI ≥ 30 kg/m^2^(total pregnancy intake between 5 and 9 kg) [24].

SGA status was defined by a birthweight < to the 10^th^ percentile according to the WHO fetal growth charts [25]. High medical risk level before pregnancy was defined as the presence of one or more of: history of cardiac disease, hypertension, diabetes, venous thrombosis, pulmonary embolism, Graves’ disease, asthma, homozygous sickle cell anemia, thrombocytopenia, coagulation disorder, a rare or systemic disease, nephropathy, HIV infection, psychiatric disease.

High obstetrical risk level before pregnancy was defined by a history of one or more of the following: pre-eclampsia, fetal growth restriction, preterm delivery, fetal death or neonatal death.

Pregnancy complication was defined as the occurrence of one or more of the following complications: gestational diabetes, pre-eclampsia, fetal growth restriction, proteinuria, thrombocytopenia, threatened preterm labor, preterm premature rupture of membranes (PPROM), deep vein thrombosis and intrahepatic cholestasis of pregnancy.

Stillbirth was defined as a fetal death occurring after 20 gestational weeks.

### Statistical analysis

Maternal, pregnancy, labor, delivery, and neonatal characteristics were compared using Chi^2^ or Fisher exact tests for categorical variables and Student’s or Wilcoxon rank sum tests for quantitative variables, as appropriate. All tests were two-sided with p-values < 0.05 defined as statistically significant. The independent association between premature birth < 37 GW, premature birth < 34 GW and neonatal composite morbidity and PPFU was tested using multiple logistic regression. Adjustment was performed on known confusion factors and the variables that differed between the PPFU and standard care group: maternal age, parity, SDI, BMI, high medical risk level before pregnancy and high obstetrical risk level before pregnancy. No multicollinearity was detected using variance inflation factor. Visual inspection of residual plots did not reveal any obvious deviations from homoscedasticity or normality. Propensity score matching was then used (Matchit package [26]) to test the association between PPFU and adverse pregnancy outcomes. Patients in the PPFU group were matched to two patients in the standard care group based on maternal age, parity, BMI, high medical risk level before pregnancy and high obstetrical risk level before pregnancy. Each of the variables included in the propensity score matching were balanced between the two groups. R software (R Development Core Team (2008), version 4.2.0) was used for all analyses.

### Ethics

This study (IRB00012437) was approved by the French “Comité d’Éthique de la recherche de l’hopital Foch) ethic committee and is part of the PRECACHIM project aiming at improving maternal and neonatal outcomes in case of maternal social vulnerability. This observational study waived the need to obtain informed consent according to the French law. Women were informed that their records could be used for the evaluation of medical practices and were allowed to opt out of these studies.

## Results

**Fig. 1:**
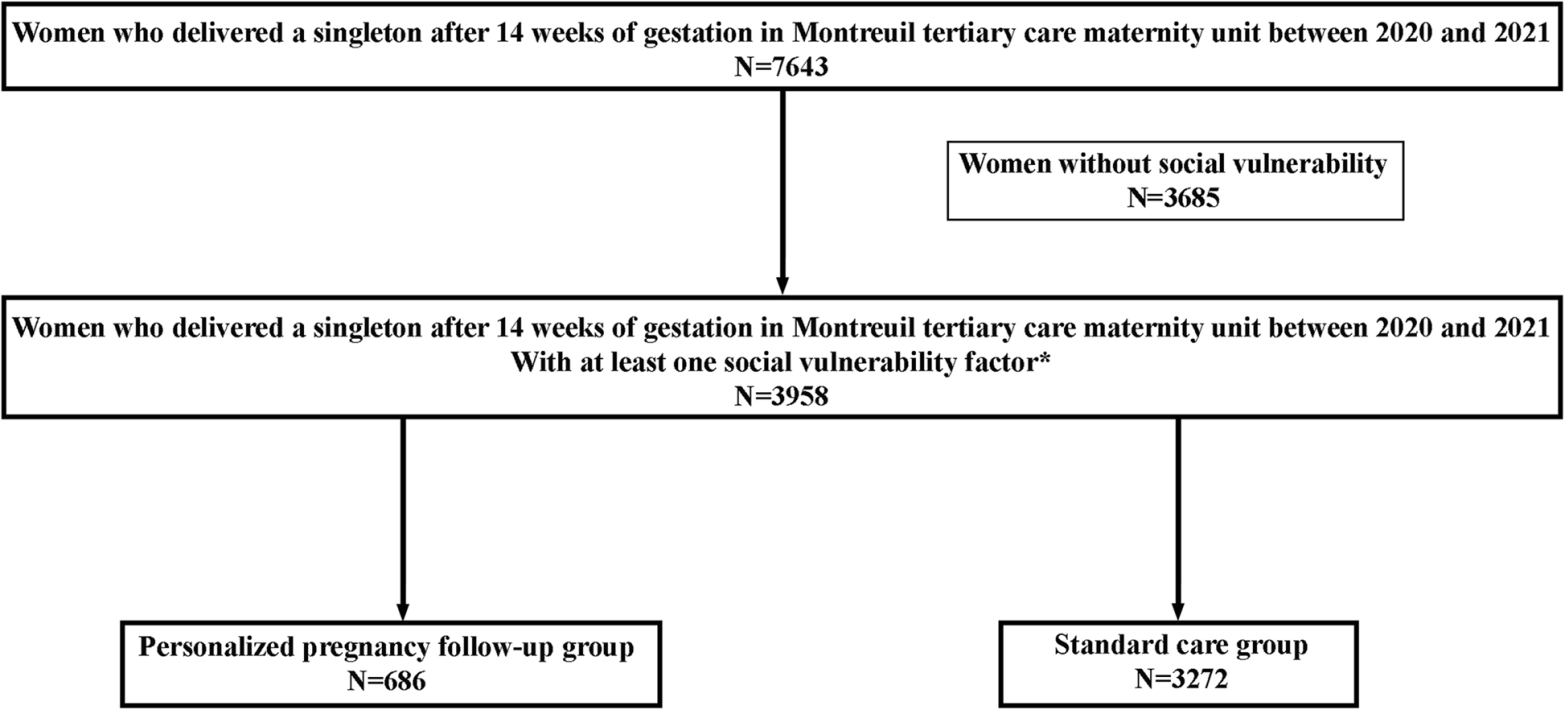
Flow Chart. *social isolation, poor or insecure housing conditions, no work-related household income, absence of standard health insurance, recent immigration (< 12 month), linguistic barrier, history of violence, being handicapped or minor, addictions

Between January 2020 and December 2021, 3958 women with social vulnerability who delivered a singleton after 14 gestational weeks were included in the study (Fig. 1): 686 in the PPFU group and 3272 in the standard care one. Patients in the PFFU group were younger and presented lower BMI (Table 1). Excessive and insufficient GWG were equally distributed between the two study groups. The PPFU group included more patients with a Sub-Saharan Africa origin. High medical risk level before pregnancy was more frequent in the PPFU group. Women included in the PPFU group presented a higher SDI with a larger number of patients presenting a SDI ≥ 3 (45% versus 19% in the standard care group, *p* < 0.001). Recent immigration, interpersonal violence during pregnancy and addictions both before and during pregnancy were more frequent in the PFFU group. Minor patients and patients with handicap were all included in the PPFU group. Linguistic barrier was more frequent in the standard care group. Patients with unwanted pregnancy were more prevalent in the PPFU group.

**Table 1:** Maternal characteristics according to study groups

	PPFU Participation		
	Yes (*N* = 686)	No (*N* = 3272)	p
**Maternal characteristics**			
Maternal age (years, mean (SD))	29.01 (6.84)	30.59 (5.72)	< 0.001
Maternal age ≥ 35 years n (%)	144 (21.0)	778 (23.8)	0.129
Parity (mean (SD))	1.36 (1.52)	1.37 (1.41)	0.909
Pre-pregnancy body mass index (kg/m^2^, mean (SD))	25.77 (5.15)	26.37 (5.57)	0.010
Maternal obesity (BMI > 30 kg/m^2^) n (%)	120 (17.5)	681 (20.8)	0.055
Gestational weight gain n (%)			0.106
Insufficient	327 (47.7)	1427 (43.6)	
Adequate	246 (35.9)	1221 (37.3)	
Excessive	113 (16.5)	624 (19.1)	
Maternal origin n (%)			< 0.001
Sub-Saharan Africa	351 (51.2)	1259 (38.5)	
Asia	67 (9.8)	389 (11.9)	
Caucasian	268 (39.1)	1624 (49.6)	
High medical risk level before pregnancy^a^ n (%)	150 (21.9)	383 (11.7)	< 0.001
High obstetrical risk level before pregnancy^b^ n (%)	166 (24.2)	909 (27.8)	0.061
**Social deprivation index**^**c**^** n (%)**			< 0.001
0	94 (13.7)	223 (6.8)	
1	102 (14.9)	1394 (42.6)	
2	181 (26.4)	1025 (31.3)	
≥ 3	309 (45.0)	630 (19.3)	
**Type of vulnerability**			
Social isolation n (%)	329 (48.0)	950 (29.0)	< 0.001
Poor or insecure housing condition n (%)	259 (37.8)	869 (26.6)	< 0.001
Not work-related household income n (%)	481 (70.1)	2040 (62.3)	< 0.001
No permanent health care insurance n (%)	432 (63.0)	1605 (49.1)	< 0.001
Recent immigration^d^ n (%)	191 (27.8)	162 (5.0)	< 0.001
Linguistic barrier n (%)	69 (10.1)	423 (12.9)	0.045
Interpersonal violence during pregnancy n (%)	373 (54.4)	173 (5.3)	< 0.001
Handicap/minor n (%)	213 (31.0)	0 (0.0)	< 0.001
Addiction before pregnancy n (%)	183 (26.7)	557 (17.0)	< 0.001
Addiction during pregnancy n (%)	156 (22.7)	423 (12.9)	< 0.001
Unwanted pregnancy n (%)	26 (3.8)	18 (0.6)	< 0.001
**Prenatal care utilization**			
Inadequate PCU^e^ n (%)	181 (26.4)	486 (14.9)	< 0.001
Term of first consultation (mean (SD))	23.99 (7.03)	24.50 (6.67)	0.073
Social worker intervention n (%)	390 (57.3)	525 (16.3)	< 0.001
Addiction therapy n (%)	38 (5.5)	51 (1.6)	< 0.001
Psychologic follow-up^f^ n (%)	393 (57.3)	365 (11.2)	< 0.001
Psychiatrist follow-up^g^ n (%)	98 (14.3)	33 (1.0)	< 0.001
Number of pregnancy consultation (mean (SD))	6.52 (5.65)	5.30 (4.05)	< 0.001
Number of hospitalization (mean (SD))	1.66 (1.46)	1.52 (1.18)	0.006
Number of Emergency consultation (mean (SD))	1.69 (1.88)	1.59 (1.79)	0.200

^a^defined as the presence of one or more of: history of cardiac disease, hypertension, diabetes, venous thrombosis, pulmonary embolism, Graves’ disease, asthma, homozygous sickle cell anemia, thrombocytopenia, coagulation disorder, a rare or systemic disease, nephropathy, HIV infection, psychiatric disease

^b^defined by a history of one or more of the following: pre-eclampsia, fetal growth restriction, preterm delivery, fetal or neonatal death

^c^simple sum of 4 deprivation dimensions: social isolation, poor or insecure housing condition, not work-related household income, and no permanent heath care insurance

^e^pregnancy follow-up began after 12 weeks of gestation, or if it included less than 50% of the number of prenatal visits expected according to duration of pregnancy, or if the first-trimester ultrasound examination or both the second- and third- trimester examinations were missing

^f^Psychologic follow-up was performed in case of pregnancy related anxiety, depressive symptoms, or patient request for a psychologist follow-up

^g^Psychiatrist follow-up was performed in case of major depressive disorder, bipolar disorder, post-traumatic stress disorder or Schizophrenia

Inadequate PCU was more frequent in the PPFU group (Table 1). Women included in the PPFU group benefitted from more social worker intervention, addiction therapy, psychologic and psychiatric follow-up compared to the standard care group. The number of pregnancy follow-up appointments and hospitalizations was higher in the PPFU group whereas emergency consultations were equally distributed between the two groups.

Pregnancy complications were similar between the two groups (Table 2) and each complication taken separately was equally distributed (Additional Table 1).

**Table 2:** Pregnancy, labor and neonatal outcomes according to study group

	PPFU Participation		
	Yes (*N* = 686)	No (*N* = 3272)	p
**Pregnancy characteristics**			
Pregnancy complication^a^ n (%)	433 (63.1)	2128 (65.0)	0.362
**Labor outcomes**			
Induction of labor n (%)	258 (37.6)	1177 (36.0)	0.443
Delivery mode n (%)			
Cesarean section before labor	57 (8.3)	193 (5.9)	0.023
Emergency Cesarean section before labor	28 (4.1)	129 (3.9)	0.95
Cesarean section during labor	77 (11.2)	314 (9.6)	0.22
Post-Partum hemorrhage n (%)	48 (7.0)	214 (6.5)	0.724
**Neonatal outcomes**			
Premature birth (< 37 weeks) n (%)	59 (8.6)	357 (10.9)	0.084
Premature birth (< 34 weeks) n (%)	29 (4.2)	209 (6.4)	0.038
Neonatal intensive care unit admission n (%)	46 (6.7)	318 (9.7)	0.016
5-min Apgar score < 7	18 (2.6)	83 (2.5)	1.000
Cord blood pH < 7.10	24 (3.5)	118 (3.6)	0.980
Small for gestational age^c^ n (%)	159 (23.2)	671 (20.5)	0.131
Pregnancy outcome n (%)			0.263
Neonatal death	1 (0.1)	1 (0.0)	
Miscarriage/abortus	2 (0.3)	17 (0.5)	
Medical abortion	2 (0.3)	23 (0.7)	
Stillbirth	3 (0.4)	30 (0.9)	

*PPFU* Personalized pregnancy follow-up

^a^defined as the occurrence of one or more of the following complications: gestational diabetes, pre-eclampsia, fetal growth restriction, proteinuria, thrombopenia, threatened preterm labor, premature rupture of membranes (PROM), deep vein thrombosis and cholestasis of pregnancy

^b^defined by a cord blood pH < 7.10 and/or a 5-min Apgar score < 7 and/or neonatal intensive care unit admission

^c^Small for gestational was defined by a birthweight < to the 10th percentile according to the WHO fetal growth charts

Induction of labor was similar between the two groups (Table 2). Women included in the PPFU group delivered more often by cesarean section before labor whereas the emergency cesarean section rates were similar between the two groups.

Regarding neonatal outcomes, the premature birth < 37 GW rate were similar between the two groups (10.9% in the standard care group versus 8.6% in the PPFU group, *p* = 0.084) whereas the PPFU group unveiled a lower rate of premature birth < 34 GW (6.4% for the standard care group and 4.2% in the PPFU one, *p* = 0.038). SGA status was equally distributed between the group groups. Women in the PPFU group had a lower rate of NICU admission (6.7% for the PPFU group versus 9.7% for the standard care group).

After adjustment on the social deprivation index, maternal age, parity, BMI, maternal origin, high medical and high obstetrical risk level before pregnancy (Table 3), PPFU was associated with a reduced risk of premature birth < 37 GW (aOR = 0.66, 95%CI[0.49–0.89]), a reduced risk of premature birth < 34 GW, (aOR = 0.53, 95%[0.34–0.79]). There was no association between PPFU and SGA (aOR = 1.06, 95%CI [0.86 – 1.30]). Propensity score matching adjusted (psa) OR performed on the same variables unveiled similar results for premature birth < 37 GW (psaOR = 0.63, 95%CI[0.46–0.86]), for premature birth < 34 GW (psaOR = 0.52, 95%CI[0.34–0.78] and for SGA status (psaOR = 1.07, 95%CI [0.86 – 1.33]).

**Table 3:** Association between PPFU inclusion and poor neonatal outcomes

	**Premature birth (< 37 weeks)**		
*N* = 3958	OR	aOR	PSaOR
PPFU	0.77 [0.57—1.02]	0.66[0.49–0.89]^**^	0.63[0.46–0.86]^**^
Social deprivation index^a^	1.08 [1.01 – 1.16]^*^	1.16[1.04–1.30]^*^	
Maternal age	1.03 [1.01 – 1.04]^***^	1.03[1.01–1.05]^**^	
Parity	1.00 [0.93—1.07]	0.91[0.83–0.98]^*^	
Pre-pregnancy body mass index (kg/m^2^)	1.01 [0.99—1.03]	0.99[0.98–1.01]	
Maternal origin			
Caucasian	ref	ref	
Asian	1.17 [0.84—1.61]	1.18[0.84–1.63]	
Sub-Saharan Africa	1.13 [0.91—1.40]	1.15[0.92–1.44]	
High medical risk level before pregnancy^b^	2.46 [1.92—3.12]^***^	2.44[1.89–3.12]^***^	
High obstetrical risk level before pregnancy^c^	1.67 [1.35—2.07]^***^	1.58[1.26–1.98]^***^	
	**Premature birth (< 34 weeks)**		
*N* = 3958	OR	aOR	PSaOR
PPFU	0.65 [0.43—0.95]^*^	0.53[0.34–0.79]^**^	0.52[0.34–0.78]^**^
Social deprivation index^a^	1.18 [1.02—1.36]^*^	1.28[1.11–1.49]^**^	
Maternal age	1.04 [1.01—1.06]^**^	1.05[1.02–1.07]^***^	
Parity	1.03 [1.01—1.05]^*^	0.77[0.68–0.86]^***^	
Pre-pregnancy body mass index (kg/m^2^)	1.04 [1.02 – 1.06]	1.02[0.99–1.04]	
Maternal origin			
Caucasian	ref	ref	
Asian	1.30 [0.84—1.96]	1.31[0.84–1.98]	
Sub-Saharan Africa	1.37 [1.04—1.82]^*^	1.47[1.10–1.97]^**^	
High medical risk level before pregnancy^b^	2.26 [1.77 – 2.86]^***^	2.45[1.77–3.34]^***^	
High obstetrical risk level before pregnancy^c^	1.83 [1.50 – 2.23]^***^	1.70[1.27–2.26]^***^	
	**Small for gestational age**^**d**^		
*N* = 3958	OR	aOR	PSaOR
PPFU	1.17 [0.96 – 1.42]	1.06 [0.86 – 1.30]	1.07 [0.86 – 1.33]
Social deprivation index^a^	1.09 [1.00 – 1.18]^*^	1.07 [0.98 – 1.16]	
Maternal age	0.99 [0.98 – 1.00]	1.01 [0.99 – 1.02]	
Parity	0.87 |0.82 – 0.92]^***^	0.87 [0.81 – 0.93]^***^	
Pre-pregnancy body mass index (kg/m^2^)	0.96 [0.95 – 0.97]***	0.96 [0.95 – 0.98]^***^	
Maternal origin			
Caucasian	ref	ref	
Asian	1.27 [0.99 – 1.63]	1.27 [0.98 – 1.62]	
Sub-Saharan Africa	1.36 [1.16 – 1.60]^***^	1.45 [1.22 – 1.71]^***^	
High medical risk level before pregnancy^b^	1.14 [0.91 – 1.41]	1.20 [0.96 – 1.50]	
High obstetrical risk level before pregnancy^c^	0.83 [0.70 – 1.00]	0.93 [0.78 – 1.12]	

*PPFU* Personalized pregnancy follow-up

*OR* Odd ratio

*aOR* Adjusted OR, adjustment on maternal age, parity, SDI, BMI, high medical risk level before pregnancy and high obstetrical risk level before pregnancy

*PSaOR* Propensity score adjusted OR, patients in the PPFU group were matched to two patients in the standard care group based on maternal age, parity, BMI, high medical risk level before pregnancy and high obstetrical risk level before pregnancy

^*^p < 0.05

^**^p < 0.01

^***^p < 0.001

^a^simple sum of 4 deprivation dimensions: social isolation, poor or insecure housing condition, not work-related household income, and no permanent heath care insurance

^b^defined as the presence of one or more of: history of cardiac disease, hypertension, diabetes, venous thrombosis, pulmonary embolism, Graves’ disease, asthma, homozygous sickle cell anemia, thrombocytopenia, coagulation disorder, a rare or systemic disease, nephropathy, HIV infection, psychiatric disease

^c^defined by a history of one or more of the following: pre-eclampsia, fetal growth restriction, preterm delivery, fetal or neonatal death

^d^Small for gestational was defined by a birthweight < to the 10th percentile according to the WHO fetal growth charts

## Discussion

To our knowledge, this retrospective study is the first to evaluate a personalized patient-based intervention to address all types of social vulnerabilities with the aim of reducing poor neonatal outcomes. This work highly suggests that PPFU reduces the impact of social stress factors on prematurity and neonatal morbidity by improving medical care access and coordination for socially deprived women.

Indeed, patients included in the PPFU group had a significantly better access to any of the different care providers such as social care workers, psychologists, and addiction therapist. Even if inadequate PCU was more frequent at patient inclusion in the PPFU group, PPFU patients had more pregnancy follow-up appointments and were more often hospitalized (Table 1). This point suggests that PPFU patients had a more appropriate pregnancy follow-up given that the rate of pregnancy complications was similar between the two groups and might explain the differences observed in terms of prematurity. Moreover, maternal psychiatric disorders [27], pregnancy related anxiety in socially deprived women [28] and maternal stress [29] are established risk factors of premature birth. Therefore, the benefit regarding prematurity in the PPFU group can be explained by the difference in terms of psychologic follow-up, psychiatric follow-up, and addiction therapy.

The novelty of our personalized clinical approach comes from the facts that PPFU addresses all types of maternal social vulnerability and proposes customized pregnancy follow-up. Former studies aiming at reducing the impact of social vulnerabilities on neonatal outcomes in the US demonstrated that reducing psychosocial stress factor along with dietary counselling [20] or improving prenatal care access [21] reduces the rate of low birth weight and prematurity in the sub-group of Hispanic women [21]. Yet, within these studies, included patients were proposed similar pregnancy follow-up and only particular types of social vulnerabilities were addressed.

This work suggests that PPFU reduces prematurity regardless of maternal ethnicity. Yet, PPFU had no impact on SGA rates compared to the two studies carried out in the US [20, 21]. This point can be explained by the absence of dietary counselling within the PPFU. Indeed, inadequate GWG was similar between the PPFU and standard care group.

It is of importance to highlight that this study was carried out in high risk patients with high figures of social vulnerability, which was also the case for the two aforementioned studies [20, 21]. Indeed, a recent randomized control trial in the Netherlands testing an intervention aiming at detecting and addressing social deprivation in a low risk population that didn’t reach the expected figures of social deprivation [30], didn’t show any benefit regarding low birth weight and premature birth.

It is of note that the results are consistent with previous studies addressing social vulnerability and poor pregnancy outcomes (Table 3). Indeed, the increase of the social deprivation index was independently associated with premature birth (< 37 GW) (aOR = 1.16[1.04–1.30] for one point increase of the SDI), premature birth (< 34GW) (aOR = 1.28[1.11–1.49]) [2, 4, 5, 7, 16, 23]. In addition, these data are consistent with the impact of PCU on pregnancy outcomes unveiled in former studies [15, 16]. Indeed, the main differences between the two study groups resides in the access to both medical and non-medical prenatal care (Table 1). This suggests that the differences observed in terms of poor neonatal outcomes comes from the improvement of prenatal care use.

The main strength of this study is the combined accurate and thorough access to both precise data on the social vulnerabilities and medical factors thanks to the computerized patient folder. This was the result of the training of all the professionals of the maternity unit performed by the PPFU unit midwifes since 2017, in the detection of social vulnerabilities. Moreover, the sample size of socially deprived women is substantial compared to former studies conducted in France [10, 15, 31]. The access to medical and obstetrical history of the patients allowed to properly adjust on medical and non-medical factors in the multiple logistic regression and propensity score matching (Table 3).

Yet, this study is not without limitations. The retrospective and unicentric design might limit the generalization of the results. Indeed, the population studied in this work presented a high level of social deprivation with 54.2% of the included patients presenting a SDI ≥ 2 and 23.7% with a SDI ≥ 3. Moreover, the neighborhood of the maternity unit at study present a high level of social deprivation with a high rate of neonatal mortality [32]. Finally, the vulnerable women population included in this study presented a high medical risk level and a high rate of pregnancy complications (Table 1, and Table 2). Therefore, the application of the same protocol in a less socially deprived population with a lower medical risk might alter the impact of PPFU. Lastly, the retrospective design of this study lessens the strength of the results. Yet, testing this intervention in a prospective randomized manner would be ethically questionable given the impact of social vulnerabilities on pregnancy outcomes.

## Conclusions

This work suggests that PPFU to address social vulnerabilities reduces prematurity and neonatal morbidity. These results could be explained by an improvement of relevant prenatal care access.

Further research should focus on characterizing the relevant non-medical needs according to social vulnerability profiles to improve both mother’s and neonates’ wellbeing.

## Supplementary Information

**Additional file 1.**

## Data Availability

The datasets used and/or analyzed during the current study are available from the corresponding author on reasonable request.

## Abbreviations

PPFU: Personalized pregnancy follow-up
SDI: Social deprivation index
SGA: Small for gestational age
PCU: Prenatal care use
BMI: Body mass index
OR: Odd ratio
aOR: Adjusted Odd ratio
PSaOR: Propensity score adjusted odd ratio
