# Supplementary material for: Use of pregnancy personalised follow-up in case of maternal social vulnerability to reduce prematurity and neonatal morbidity
Source: BMC Pregnancy Childbirth. 2023 Apr 26;23:289. doi: 10.1186/s12884-023-05604-7 (PMC10131299; doi:10.1186/s12884-023-05604-7)
Supplement: Supplementary file 1 — Additional file 1. [file 12884_2023_5604_MOESM1_ESM.docx]

**Additional Table 1: Pregnancy complications according to study groups**

|  | PPFU Participation | |  |
| --- | --- | --- | --- |
|  | Yes (N=686) | No (N=3272) | p |
| **Pregnancy complications** |  |  |  |
| Gestational diabetes n (%) | 170 (24.8) | 876 (26.8) | 0.304 |
| Gestational diabetes requiring insulin n (%) | 49 (7.1) | 226 (6.9) | 0.890 |
| Gestational hypertension n (%) | 69 (10.1) | 278 (8.5) | 0.215 |
| Preeclampsia n (%) | 37 (5.4) | 183 (5.6) | 0.908 |
| Proteinuria n (%) | 154 (22.4) | 692 (21.1) | 0.482 |
| Fetal growth restriction n (%) | 65 (9.5) | 310 (9.5) | 1.000 |
| Cholestasis n (%) | 15 (2.2) | 76 (2.3) | 0.939 |
| Threatened preterm labor n (%) | 34 (5.0) | 166 (5.1) | 0.975 |
| PPROM^a^ n (%) | 51 (7.4) | 254 (7.8) | 0.830 |
| Thrombopenia n (%) | 24 (3.5) | 122 (3.7) | 0.858 |
| Deep vein thrombosis n (%) | 15 (2.2) | 91 (2.8) | 0.455 |

PPFU: Personalized pregnancy follow-up

^a^Preterm premature rupture of membranes
